# Temperature Significantly Affects the Plaquing and Adsorption Efficiencies of *Listeria* Phages

**DOI:** 10.3389/fmicb.2016.00631

**Published:** 2016-05-03

**Authors:** Jeffrey I. Tokman, David J. Kent, Martin Wiedmann, Thomas Denes

**Affiliations:** Department of Food Science, Cornell University, IthacaNY, USA

**Keywords:** bacteriophages, *Listeria monocytogenes*, food safety, teichoic acids, phage-resistance, physiological refuge

## Abstract

*Listeria*-infecting phages are currently being used to control and detect the important foodborne pathogen *Listeria monocytogenes*; however, the influence of environmental conditions on the interactions between *L. monocytogenes* and its phages has not been explored in depth. Here, we examined the infective potential of four *Listeria* phages (two each from the P70-like and P100-like phages of *Listeria*) against five strains of *L. monocytogenes* (representing serotypes 1/2a, 1/2b, 4a, and 4b) grown under a range of temperatures (7–37°C). We show that the plaquing efficiencies for all four phages were significantly affected by temperature. Interestingly, no plaques were observed for any of the four phages at 37°C. Adsorption assays performed with the P100-like phages, LP-048 and LP-125, showed that LP-048 had a severely reduced adsorption efficiency against susceptible strains at 37°C as compared to 30°C, suggesting that there is considerably less accessible rhamnose (LP-048’s putative phage receptor) on the host at 37°C than at 30°C. LP-125 adsorbed to host cells at 37°C, indicating that the inability for LP-125 to plaque at 37°C is not due to adsorption inhibition. LP-048 showed significantly higher adsorption efficiency against a mutant strain lacking *N*-acetylglucosamine in its wall teichoic acids (WTA) than the parental strain at both 30 and 37°C, suggesting that *N*-acetylglucosamine competes with rhamnose for glycosylation sites on the WTA. The data presented here clearly shows that *L. monocytogenes* can gain physiological refuge from phage infection, which should be carefully considered for both the design and implementation of phage-based control and detection applications.

## Introduction

*Listeria monocytogenes* is the foodborne pathogen that causes listeriosis, which has a mortality rate of 20–30% (Vazquez-Boland et al., 2001) and causes an estimated 255 deaths per year in the US (Scallan et al., 2011). It has been estimated that the total annual cost of *L. monocytogenes* in the US is ∼$2.5 billion (Batz et al., 2012). A recent meta-analysis on the global burden of listeriosis found that there is an estimated 23,150 human incidents of listeriosis every year (de Noordhout et al., 2014). Outbreaks caused by *L. monocytogenes* are a major concern for the food industry; between 1998 and 2008 there have been 24 confirmed outbreaks of listeriosis in the US, which resulted in a total of 359 illnesses, 215 hospitalizations and 38 deaths (Cartwright et al., 2013).

Since 2006, lytic bacteriophages (or “phages”) have been used according to national or regional regulations as biocontrol agents that specifically target *L. monocytogenes.* Studies that have evaluated the efficacy of phage-based biocontrol of *L. monocytogenes* have shown promise for this listeriocidal application (Strydom and Witthuhn, 2015); however, several obstacles still need to be overcome to improve the long-term efficacy of phage-based biocontrol of *L. monocytogenes.* For example, the influence of environmental conditions on the susceptibility of *L. monocytogenes* to phage infection has not been explored in depth (Denes and Wiedmann, 2014). A study by Kim and Kathariou (2009) showed that typically phage-sensitive epidemic clone II strains of *L. monocytogenes* were broadly resistant to phage infection at temperatures below 30°C. A follow-up study found that the temperature-dependent phage resistance was due to a restriction modification system that was expressed at lower temperatures (Kim et al., 2012); however, it is not known if other strains have similar temperature dependent mechanisms of phage-resistance.

Here, we examined how temperature affects the infection potential of *Listeria*-infecting phages against a representative panel of *L. monocytogenes* strains. Our goal was to advance our understanding of how environmental conditions affect phage-susceptibility of *Listeria* by focusing on the key condition, temperature. Further, this study was designed to provide knowledge that can improve the implementation and design of phage-based biocontrol and detection applications.

## Materials and Methods

### Phages and Bacterial Strains

Two phages each from the obligate lytic phage groups P100-like phages (phages LP-048 and LP-125) and P70-like phages (LP-026 and LP-037) were selected for use in this study (Denes et al., 2014) [**Table 1**]. We also included *Listeria* phage A511 (**Table 1**) so that we could make comparisons to other studies. The *L. monocytogenes* strains used in this study were selected to represent a diversity of lineages (Lineage I, II, and IV) and serotypes (1/2a, 1/2b, 4a, and 4b; **Table 1**). The P100-like phages used in this study were propagated on Mack and the P70-like phages used in this study were propagated on FSL J1-208 (also the isolation host) as previously described (Vongkamjan et al., 2012). The plasmid cured strain of FSL J1-208, designated FSL B2-294, was also included in this study to test whether the plasmid is linked to phage susceptibility (**Table 1**). The strains 541-NM, 542-NM, and ΔdltA (**Table 1**) were used in this study because the changes to their surface structures are known to affect (Denes et al., 2015), or may affect, phage adsorption. Strains 541-NM-C and 542-NM-C were included (**Table 1**) to show that complementation of the mutations in 541-NM and 542-NM with the wild-type alleles of LMRG_00541 and LMRG_00542, respectively, rescues the wild-type phenotype.

**Table 1:** Phages and bacterial strains used in study.

Strain or Phage	Alternative ID^∗^	Description	Reference
***L. monocytogenes***			
***strains***			
10403S		Lineage II, Serotype 1/2a	Bishop and Hinrichs, 1987
Mack		Lineage II, Serotype 1/2a	Hodgson, 2000
FSL J1-175	J1-175	Lineage I, Serotype 1/2b	Bergholz et al., 2010; Stasiewicz et al., 2010
F2365		Lineage I, Serotype 4b	Linnan et al., 1988; Wesley and Ashton, 1991
FSL J1-208	J1-208	Lineage IV, Serotype 4a	den Bakker et al., 2012
FSL B2-294	B2-294	J1-208 cured of pLMIV	den Bakker et al., 2012
FSL D4-0014	541-NM	Nonsense mutation (NM) in LMRG_00541; 10403S background; GlcNAc**^-^**^†^	Denes et al., 2015
FSL D4-0161	541-NM-C	541-NM complemented (C) with WT allele of LMRG_00541	Denes et al., 2015
FSL D4-0119	542-NM	Nonsense mutation (NM) in LMRG_00542; 10403S background; Rha**^-^**^‡^	Denes et al., 2015
FSL D4-0156	542-NM-C	542-NM complemented (C) with WT allele of LMRG_00542	Denes et al., 2015
Hel-867	ΔdltA	Deletion mutation (Δ) of LMRG_02073 (*dltA*); 10403S background	Gift from H. Marquis
***Phages***			
LP-026		P70-like phage	Vongkamjan et al., 2012; Denes et al., 2014
LP-037		P70-like phage	Vongkamjan et al., 2012; Denes et al., 2014
LP-048		P100-like phage	Vongkamjan et al., 2012; Denes et al., 2014
LP-0125		P100-like phage	Vongkamjan et al., 2012; Denes et al., 2014
A511		P100-like phage	Loessner and Busse, 1990

*^∗^Strain ID used in the manuscript text and figures. ^†^The wall teichoic acids of the indicated strain lack *N*-acetylglucosamine (GlcNAc). ^‡^The wall teichoic acids of the indicated strain lack rhamnose (Rha).*

### Efficiency of Plaquing Assays

Bacterial lawns were prepared using a double agar overlay method (Kropinski et al., 2009) using modified LB MOPS media (at a pH of 7.4) as previously described (Vongkamjan et al., 2012). A 30 μl aliquot of *L. monocytogenes* culture grown overnight (16 ± 2 h) in BHI broth (shaking at 210 RPM) was used to seed each lawn. Immediately after bacterial lawns were poured the plates were acclimated for 60 to 90 min at the experimental temperatures before phage dilutions were spotted on the acclimated lawn. Phage dilutions that were spotted on plates ranging in concentration from 2 × 10^2^ to 3 × 10^9^PFU/mL. Immediately after spotting, the plates were incubated at the experimental temperatures. Temperatures were selected to represent conditions phage-based products would be expected to function under; for example, 6.5°C is a temperature that some refrigerated foods may be held at, and is similar to the 7°C temperature used by Kang et al. (2014) in *Listeria* growth studies on cold-smoked salmon. Plates incubated at 21 ± 1, 25 ± 1, 30 ± 2, and 37 ± 1°C were incubated for 16 ± 2 h before enumeration. Plates incubated at 12 ± 1 and 16.5 ± 1.5°C were incubated for 40 ± 2 h before enumeration. Plates incubated at 6.5 ± 1.5°C were incubated for 7 days before enumeration. Longer incubation times at lower temperatures were needed to allow for visible plaques to form. Only visible plaques were counted; faint turbidity without plaque formation was scored as zero.

### Adsorption Assays

LP-048 and LP-125 adsorption assays were performed by following a modified version of a previously described procedure (Denes et al., 2015). Modifications were made to adapt the procedure to compare adsorption at different experimental temperatures (30°C vs. 37°C). Briefly, overnight cultures (incubated for 16 ± 2 h) were grown in BHI broth at the experimental temperatures with shaking at 210 RPM and then diluted 1/100 into fresh BHI broth. The diluted cultures were then incubated at their respective experimental temperature (30°C or 37°C) with shaking at 210 RPM until they reached an OD_600_ of 0.4. Then, 200 μl volumes of cultures (at an OD_600_ value of 0.4) were transferred into microcentrifuge tubes containing 762 μl BHI broth, 20 μl phage lysate at 1 × 10^9^PFU/mL, 9 μl of 1M CaCl_2_, and 9 μl of 1M MgCl_2_ (salts were added immediately prior to the addition of bacteria). The bacteria and phage mixture was then incubated for 15 min at either 30°C or 37°C with aeration.

### Statistical Analyses

All statistical analyses were carried out in R (V.3.0.3; R Core Team, 2009). Data handling was performed using reshape (Wickham, 2007). Linear models were constructed using the lm command from the base package. Pairwise comparisons at all factor levels were made using lsmeans with Tukey multiple testing correction (Lenth and Hervé, 2014). The cutoff for significance was set at α = 0.05.

Models for efficiency of plaquing (EOP) experiments and models for adsorption assay experiments had the factors strain, temperature, and biological replicate, as well as the 2-way interaction between strain and temperature. Relative EOP values were the model responses for plaquing experiments, whereas the log_10_ reductions of PFU in the supernatant were the model responses for adsorption assays.

### BLAST Searches

BLAST searches (Altschul et al., 1990) were conducted against the *L. monocytogenes* 10403S (GenBank accession no. NC_017544.1), F2365 (GenBank accession no. NC_002973.6), and J1-208 (GenBank accession no. NZ_CM001469.1) genomes with the protein query sequences for the LmoH7 restriction–modification system (GenBank accession nos. EAL08857.1 and EAL08858.1). We defined our cutoff for homology as 25% shared sequence identity with ≥50% query coverage.

### Data and Script Availability

The raw data and R scripts used in this study can be found in the Supplementary File 1.

## Results

### Spot Assays Reveal That Temperature Affects Phage Infection of *L. monocytogenes*

**FIGURE 1:**
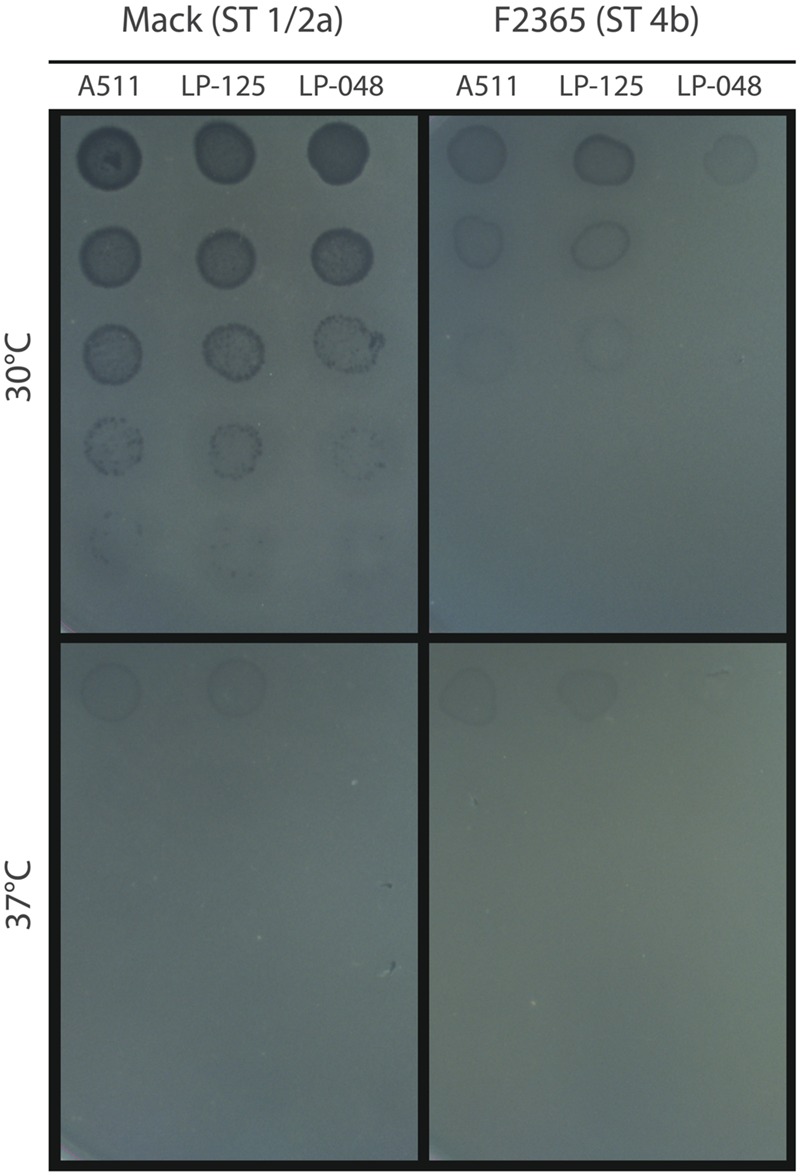
**Spot assays of phage dilutions at 30 and 37°C.** Dilutions of phages A511, LP-125, and LP-048 were spotted on lawns of Mack and F2365, which were pre- and post-incubated at the temperature indicated in the figure. Phage dilutions decrease from top (most concentrated; ∼2 × 10^7^PFU/mL) to bottom of the figure (least concentrated; ∼2 × 10^3^PFU/mL). Each dilution was 1/10.

Initial spot testing of phages A511, LP-125, and LP-048 revealed no visible plaques on Mack or F2365 at 37°C, although faint clearings were observed where the most concentrated phage solutions were spotted (**Figure 1**). All three phages formed visible plaques on Mack at 30°C (**Figure 1**); however, only LP-125 and A511 formed visible plaques on F2365 at 30°C. The EOP of LP-125 and A511 at 30°C was lower on F2365 than on Mack (**Figure 1**).

### Efficiency of Plaquing of P100-like Phages Is Affected by Growth Temperature

**FIGURE 2:**
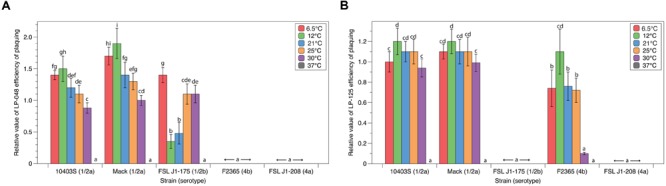
**Plaquing efficiencies of P100-like phages at varying growth temperatures.** The relative number of visible plaques under different strain and temperature conditions were compared for phages **(A)** LP-048 and **(B)** LP-125. All values are relative to the phages isolation conditions (strain Mack at 30°C). Bars that do not share any letters represent values that are significantly different; for example, a bar marked with d, e, and f is significantly different from a bar marked with g and h. Three biological replicates were conducted. Error bars represent the standard error.

Quantitative plaquing assay data collected at six different temperatures with five different host strains and two different P100-like phages (LP-048 and LP-125) were used to statistically evaluate the effects of host and temperature on plaquing. For LP-048, plaquing efficiency was significantly affected by both strain (*p* < 0.001) and temperature (*p* < 0.001); there was also a significant interaction between strain and temperature (*p* < 0.001; see Supplementary File 1). LP-048 formed visible plaques on 10403S, Mack, and J1-175 at all temperatures except 37°C, but did not form visible plaques on F2365 and J1-208 at any temperature (**Figure 2A**). Effects of temperature on plaque formation were similar for 10403S and Mack; LP-048 showed a trend of having a higher EOP at lower temperatures (**Figure 2A**) with numerically largest EOPs at 12°C and significantly lower EOPs at higher temperatures for both serotype 1/2 host strains (**Figure 2A**). Interestingly, temperature dependent patterns of plaque formation on the host strain J1-175 were different; on this host strain, the EOP values of LP-048 were significantly lower at 12 and 21°C as compared to the EOP values at 6.5, 25, and 30°C (**Figure 2A**). This may suggest there is a mechanism of phage-resistance in J1-175 (e.g., a restriction modification system) that is only expressed at temperatures between 12 and 21°C. This would be consistent with the previously described temperature-dependent restriction modification system found in epidemic clone II strains of *L. monocytogenes*, which is only active at temperatures below 30°C (Kim et al., 2012).

For LP-125, plaquing efficiency was significantly affected by both strain (*p* < 0.001) and temperature (*p* < 0.001); there was also a significant interaction between strain and temperature (*p* < 0.001; see Supplementary File 1). LP-125 formed visible plaques on 10403S, Mack, and F2365 at all temperatures except 37°C, but did not form visible plaques on J1-175 and J1-208 at any temperature (**Figure 2B**). Effects of temperature on plaque formation were similar for 10403S and Mack; LP-125 showed a trend of having a higher EOP at lower temperatures (**Figure 2B**) with numerically largest EOPs at 12°C and significantly lower EOPs at 30°C for both host strains (**Figure 2B**). Interestingly, temperature dependent differences in plaque formation were more pronounced on F2365; on this host strain the EOP was significantly higher at 12°C (as compared to all other temperatures), and the EOP was significantly lower at 30°C than at temperatures between 6.5 and 25°C (**Figure 2B**).

### Efficiency of Plaquing of P70-like Phages Is Affected by Growth Temperature

**FIGURE 3:**
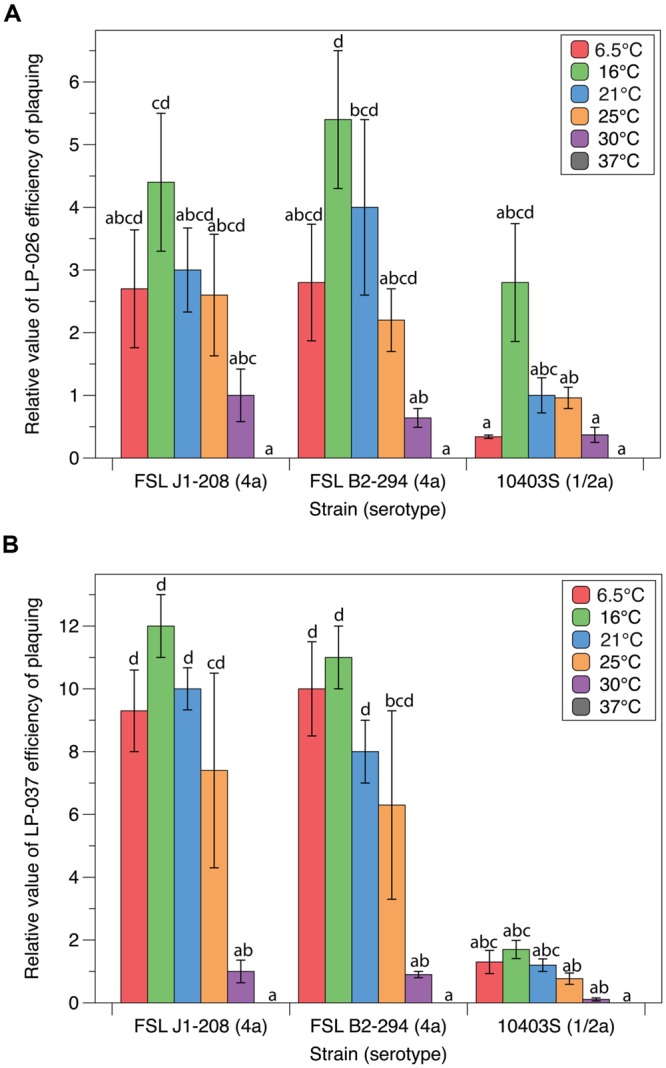
**Plaquing efficiencies of P70-like phages at varying growth temperatures.** The relative number of visible plaques under different strain and temperature conditions were compared for phages **(A)** LP-026 and **(B)** LP-037. All values are relative to the phage’s isolation conditions (strain J1-208 at 30°C). Bars that do not share any letters represent values that are significantly different; for example, a bar marked with a, b, and c is significantly different from a bar marked with d. Three biological replicates were conducted. Error bars represent the standard error.

Quantitative plaquing assay data collected at six different temperatures with three different host strains and two different P70-like phages (LP-026 and LP-037) were used to statistically evaluate the effects of host and temperature on plaquing. Host strains Mack, J1-175, and F2365 were not used because P70-like phages have a narrow host range as compared to P100-like phages (Vongkamjan et al., 2012). For LP-026, plaquing efficiency was significantly affected by both strain (*p* < 0.001) and temperature (*p* < 0.001; see Supplementary File 1). LP-026 formed visible plaques on J1-208, B2-294, and 10403S at all tested temperatures except at 37°C (**Figure 3A**). Effects of temperature on plaquing were similar for J1-208 and B2-294; LP-026 showed a general trend of infecting at a higher EOP at lower temperatures, with the numerically highest EOP at 16°C for both strains, and significantly lower EOPs at 30°C than at 16°C for B2-294 (**Figure 3A**). On strain 10403S, the EOP of LP-026 was numerically highest at 16°C; however, no significant differences were observed between the tested temperatures (**Figure 3A**).

For LP-037, plaquing efficiency was significantly affected by both strain (*p* < 0.001) and temperature (*p* < 0.001); there was also a significant interaction between strain and temperature (*p* < 0.001; see Supplementary File 1). LP-037 formed visible plaques on J1-208, B2-294, and 10403S at all tested temperatures except for 37°C (**Figure 3B**). Effects of temperature on plaquing were similar for J1-208 and B2-294; LP-037 showed a general trend of infecting at a higher EOP at lower temperatures, with the numerically highest EOP at 16°C, and significantly lower EOPs at 30°C than at 16°C for each strain (**Figure 3B**). On strain 10403S, the EOP of LP-037 was numerically highest at 16°C; however, no significant differences were observed between the tested temperatures (**Figure 3B**).

Neither LP-026 nor LP-037 showed any significant differences in EOP between J1-208 and the plasmid cured strain of J1-208, B2-294 (**Figure 3**). This suggests that the plasmid harbored by J1-208, which is unique amongst *L. monocytogenes* in gene content (den Bakker et al., 2012), does not have an effect on the EOP of the P70-like phages.

### Adsorption Efficiencies of LP-048 and LP-125 Are Affected by Temperature

**FIGURE 4:**
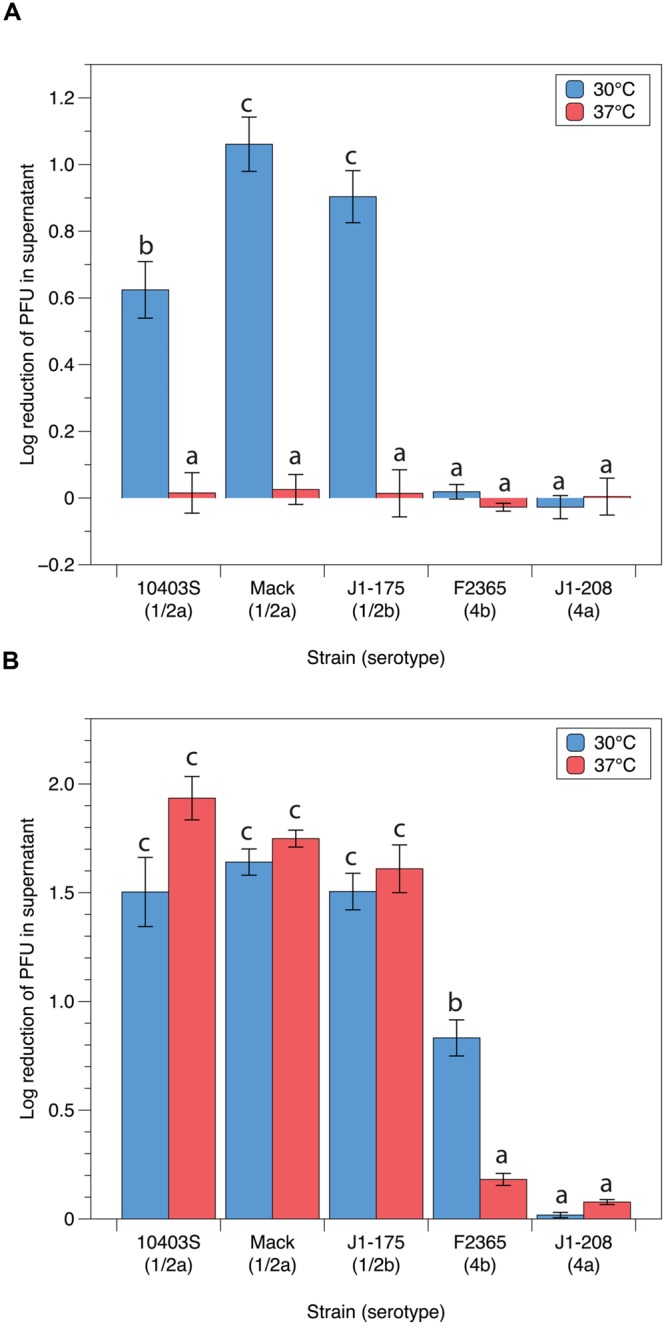
**Adsorption efficiencies of P100-like phages **(A)** LP-048 and **(B)** LP-125 at 30°C and 37°C.** Values shown are the log_10_ reduction of free phage in the supernatant of each co-incubation between phage and host. The log_10_ reduction is calculated by subtracting the log-transformed number of phage left in in the supernatant from the log-transformed number of phage in the negative control (BHI). Lower values represent less efficient adsorption. Bars that do not share any letters represent values that are significantly different; for example, a bar marked with b is significantly different from a bar marked with c. Three biological replicates were conducted. Error bars represent the standard error.

As no visible plaque formation was observed at 37°C, we set out to determine whether adsorption was inhibited at this temperature. For these experiments, we used the P100-like phages LP-125 and LP-048 as we have previously identified their putative binding receptors (Denes et al., 2015). The adsorption assays showed, based on the log_10_ reduction of free phage after co-incubation with bacteria, that LP-048 had significantly lower adsorption efficiencies at 37°C than at 30°C (0.01 to 0.03 vs. 0.62 to 1.1 log reduction) on the strains observed to be susceptible to LP-048 (10403s, Mack, and J1-175; **Figure 4A**). We did not observe LP-048 adsorption at 30°C or 37°C on the strains where LP-048 plaquing was not seen (J1-208 and F2365; **Figure 4A**). Interestingly, LP-125 showed nearly the same adsorption efficiencies at both 30 and 37°C on the serotype 1/2 strains 10403s, Mack, and J1-175; however, the adsorption efficiency of LP-125 on strain F2365 was significantly lower at 37°C than at 30°C (0.18 vs. 0.83 log reduction; **Figure 4B**). It should also be noted that LP-125 did adsorb to the strain J1-175 (**Figure 4B**), despite not forming any visible plaques on the strain (**Figure 2B**). This suggests that a phage resistance mechanism other than adsorption inhibition is preventing LP-125 from successfully infecting strain J1-175. Interestingly, this resistance mechanism is not effective against LP-048, which shares a high nucleotide similarity to LP-125 (Denes et al., 2014).

### Adsorption Efficiency of LP-048 Is Increased in a Mutant Strain Lacking GlcNAc As a Wall Teichoic Acid Sugar

**FIGURE 5:**
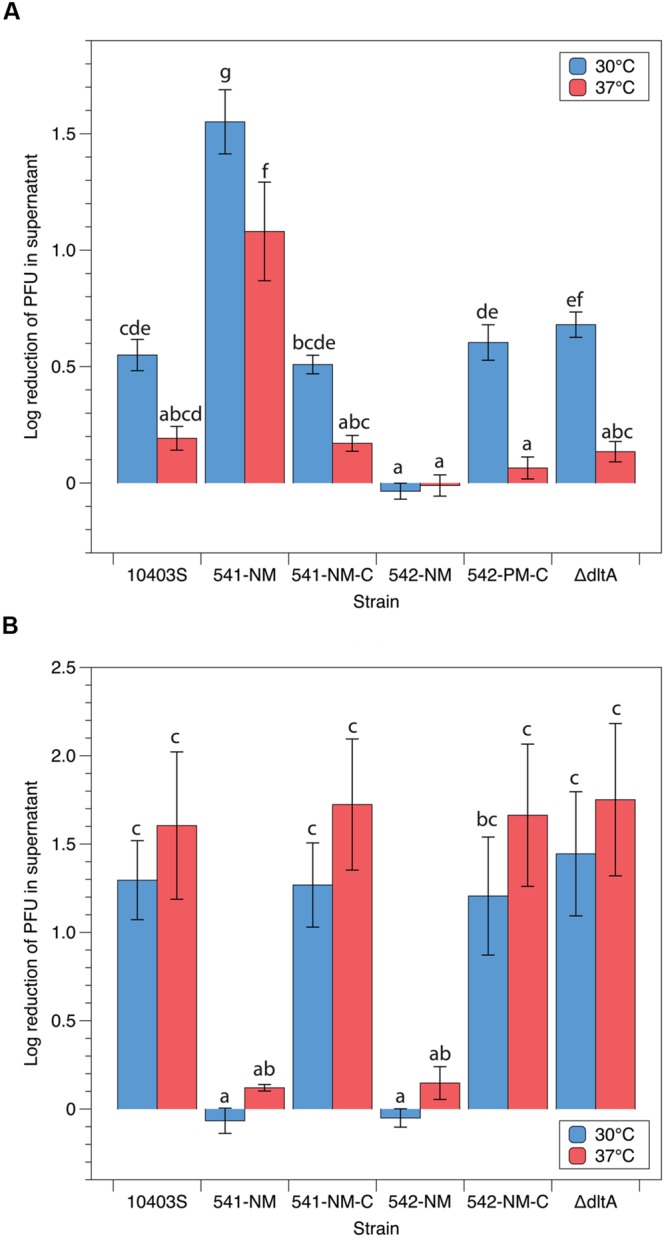
**Adsorption efficiencies of P100-like phages **(A)** LP-048 and **(B)** LP-125 on teichoic acid component mutants.** Values shown are the log_10_ reduction of free phage in the supernatant of each co-incubation between phage and host. The log_10_ reduction is calculated by subtracting the log-transformed number of phage left in in the supernatant from the log-transformed number of phage in the negative control (BHI). We also tested the ΔdltA strain complemented with the WT allele of *dltA*; however, no significant differences from either the ΔdltA strain or WT 10403S were observed. Lower values represent less efficient adsorption. Bars that do not share any letters represent values that are significantly different; for example, a bar marked with c, d, and e is significantly different from a bar marked with f. Three biological replicates were conducted. Error bars represent the standard error.

LP-048 requires rhamnose biosynthesis genes in its host for adsorption (Denes et al., 2015), these genes have since been confirmed to be essential for rhamnosylation of wall teichoic acids (WTA; Carvalho et al., 2015). We thus formulated two competing hypotheses: (i) that down-regulation of rhamnose expression on the WTA is responsible for the reduced adsorption efficiency of LP-048 observed at 37°C, or (ii) that up-regulation of GlcNAc expression on the WTA is responsible for the reduced adsorption efficiency of LP-048 observed at 37°C [as GlcNAc likely competes for the same sites on the polyol phosphate backbone of the WTA (Fiedler, 1988)]. We show here that LP-048 still adsorbs more efficiently against the mutant strain without GlcNAc in its WTA (541-NM) at 30°C than at 37°C (no significant difference of the effect of temperature between 10403S and 541-NM). This supports the first hypothesis that rhamnosylation of WTA is likely being directly down-regulated at 37°C. We also show that LP-048 does adsorb significantly more efficiently against the 541-NM mutant than against the parental strain at both 30°C (1.55 vs. 0.55 log reduction) and 37°C (1.08 vs. 0.19 log reduction; **Figure 5A**). This suggests that there is competition between rhamnose and *N*-acetylglucosamine glycosylation sites on the WTA (discussed further below); however, our data do not support that up-regulation of GlcNAc expression on the WTA is responsible for the reduced adsorption efficiency of LP-048 at 37°C. Consistent with our previous results (Denes et al., 2015), LP-048 and LP-125 showed severely reduced adsorption to the rhamnose deficient WTA mutant, 542-NM, and LP-125 showed severely reduced adsorption to 541-NM (**Figure 5**). Complementation of 541-NM and 542-NM with their respective wild-type alleles (i.e., LMRG_00541 and LMRG_00542) restored the strains’ observed phenotypes to those of the respective parental strain (10403S; **Figure 5**). We also tested adsorption of phages LP-048 and LP-125 at 30 and 37°C against the strain ΔdltA, which is incapable of incorporating D-alanine onto its teichoic acids; no significant differences were observed between ΔdltA and its parental strain (**Figure 5**).

### No Homologous Genes to LmoH7 Were Found in the Host Strains Used in This Study

Our observation that LP-125 does still adsorb at 37°C suggests that there is an adsorption-independent mechanism of phage resistance that is preventing plaque formation at 37°C. We performed BLAST searches against the genomes of strains 10403S, J1-208, and F2365 (the host strains with available genome sequences) to see if any genes homologous to the temperature dependent LmoH7 restriction–modification system found in epidemic clone II strains of *L. monocytogenes* (Kim et al., 2012) were present. No homology was found, which suggests that the adsorption-independent mechanism of phage resistance we have observed in this study is different from that caused by LmoH7.

## Discussion

In this study, we evaluated the effect of temperature on the infection process of four *Listeria* phages, representing two distinct phage groups. We showed that (i) EOP is significantly affected by temperature, most notably that no plaques were observed under any phage-host combination at 37°C, that (ii) adsorption efficiency is affected by temperature in specific cases, but does not explain the general lack of observed plaque formation at 37°C, and that (iii) there is competition between rhamnose and GlcNAc glycosylation of the WTA at both 30 and 37°C (which affects phage adsorption). Our data clearly showed that *L. monocytogenes* can gain physiological refuge from phage infection (i.e., transient resistance), which should be carefully considered when selecting combinations of phages for use in biocontrol or detection applications (Denes and Wiedmann, 2014). These data are practically important as phage-based applications for *Listeria* may need to function under a range of temperatures; for example, biocontrol tools used in food safety will likely need to work at both refrigeration and ambient temperatures, and detection assays may be expected to function under standard laboratory growth temperatures for *Listeria* (e.g., 30 and 37°C).

### Temperature Affects the Efficiency of Plaquing of P100-like Phages and P70-like Phages

Here, we showed that the efficiencies of plaquing (EOP) of phages infecting *L. monocytogenes* are affected by temperature; these results are consistent with those found in other phage-host systems. For example, Sanders and Klaenhammer (1984) found that the EOP of the *Streptococcus* phage Φ18 was significantly affected by temperature, and specifically, showed that the growth temperature of the bacteria prior to infection had more of an effect on EOP than the incubation temperature post-infection. In another study, McConnell and Wright (1979) showed that *Salmonella* phage Felix-01 showed a higher EOP on a non-sensitive strains at incubation temperatures of 23 and 30°C than at 34, 38, and 42°C. However, Ellis and Delbrück (1939) did not find any significant differenced in the EOP of an *Escherichia* phage under different growth temperatures (37, 24, and 10°C); although, unlike in the experiments here and by Sanders and Klaenhammer (1984), Ellis and Delbrück (1939) did not acclimate their host bacteria to the experimental temperature prior to exposure to the phage.

Previously, Kim and Kathariou (2009) showed that *L. monocytogenes* Epidemic Clone II strains were resistant to phage infection at temperatures below 37°C and that phages A511 and 20422-1 were active on other *L. monocytogenes* strains (including serotype 1/2a, 1/2b, 1/2c, and 4b strains) at 37°C. In contrast, A511 did not form visible plaques at 37°C under the specific conditions used in our study (**Figure 1**). In a follow-up study, Kim et al. (2012) found that a restriction modification system was expressed at temperatures below 37°C, and that deleting this restriction modification system restored phage susceptibility at 25°C. In this study, we showed a very different pattern of temperature affecting the plaquing efficiency of *L. monocytogenes.* None of the phages tested in this study formed visible plaques at 37°C, and all of the phages showed the highest EOP at temperatures under 21°C. These results were consistent with much earlier observations that 37°C incubation temperature had a deactivating effect on temperate *Listeria* phages (Audurier et al., 1977). One possible explanation for the differences observed between our study and the study by Kim et al. (2012) could be that the phages we used were all isolated from silage in New York State (USA), whereas A511 and 20422-1 were isolated from sewage and a turkey processing plant, respectively. Therefore, the phages from silage may have adapted to cooler conditions than phages A511 and 20422-1 (silage is typically stored outdoors, and New York is a temperate climate); however, as we observed here that A511 showed the same plaquing phenotype as LP-125 against Mack and F2365 at 30 and 37°C (i.e., no plaquing was observed at 37°C), future studies will be needed to test this.

### Adsorption Inhibition May Prevent Plaquing of LP-048, But Not of LP-125, at 37°C

Previously, both LP-048 and LP-125 were shown to have severe adsorption defects to *L. monocytogenes* strains with deleterious mutations in genes that are essential for rhamnosylation of WTA, which suggested that both phages use rhamnose as a phage receptor. However, LP-125 was shown to also require terminal *N*-acetylglucosamine (GlcNAc) in the WTA for successful host adsorption (Denes et al., 2015). Thus, GlcNAc and rhamnose are likely the phage receptors for LP-125 on serotype 1/2a strains, which is consistent with phages A511 (Habann et al., 2014) and P35 (Bielmann et al., 2015), both of which bind to GlcNAc and rhamnose. However, it should be noted that conclusions from Habann et al. (2014) differ from those from a prior study that identified peptidoglycan as A511’s receptor (Wendlinger et al., 1996); this inconsistency is presumably due to limitations in the methodology used by Wendlinger et al. (1996) such as relying on cell fractions, that might have different structures exposed than those found on the surface of intact cells, to assess phage adsorption. Our observation that LP-048 has a severely reduced adsorption efficiency to *L. monocytogenes* at 37°C suggests that rhamnose expression in the WTA is down regulated at 37°C. However, as LP-125 is still able to bind to *L. monocytogenes* at 37°C with similar efficiency as at 30°C, there is likely still sufficient rhamnose present at 37°C to facilitate LP-125 binding to GlcNAc residues. Furthermore, our observation that LP-125 does still adsorb at 37°C suggests that there is an adsorption-independent mechanism of phage resistance that is preventing plaque formation at 37°C. Whereas, we found no homologous genes to the previously described temperature dependent restriction–modification system LmoH7 (Kim et al., 2012), it is possible that a restriction–modification system not homologous to LmoH7 is present in the strains tested in this study. Alternatively, there could be an unrelated temperature dependent phage-defense system such as an abortive-infection system, CRISPR–Cas system, or super-infection exclusion system (Labrie et al., 2010).

We also showed that LP-125 could adsorb to the serotype 4b strain F2365 at 30°C, although significantly less efficiently than to serotype 1/2 strains. This is likely because serotype 4b strains do not possess rhamnose or terminal GlcNAc in their WTA. Instead, the WTA of serotype 4b strains are decorated with terminal glucose and galactose (Fiedler et al., 1984; Fiedler, 1988), whereas the WTA of serotype 4a strains are only decorated with terminal galactose (Fujii et al., 1985). As LP-125 binds to serotype 4b strain F2365 but not to the serotype 4a strain J1-208, we hypothesize that glucose could be the phage receptor for LP-125 on serotype 4b strains. Interestingly, we also showed that LP-125 has a significantly reduced adsorption efficiency on F2365 at 37°C, as compared to 30°C. This suggests that the surface receptor used by LP-125 on the serotype 4b strain F2365 is either down regulated or less accessible at 37°C.

### There Is Likely Competition between Rhamnose and GlcNAc Glycosylation of the WTA at Both 30 and 37°C

Our observation that the mutant strain lacking GlcNAc in its WTA (541-NM) allowed for significantly greater adsorption of LP-048 at both 30 and 37°C suggests that there is competition between the glycosylation of the WTA with rhamnose and GlcNAc. Both glycosyl groups are linked to the WTA from the same hydroxyl sites on the polyol phosphate backbone (Fiedler, 1988). The mutant strain lacking GlcNAc WTA decorations would have more hydroxyl groups in the WTA available for rhamnosylation, which would explain the increased adsorption of LP-048 against this mutant strain. Given that we still see a significant difference in adsorption of LP-048 against 541-NM between 30 and 37°C, we can rule out that the effect of temperature on LP-048 adsorption is solely due to differential regulation of GlcNAc glycosylation of the WTA; down-regulation of GlcNAc at 37°C is not increasing rhamnosylation of WTA at 37°C. It is much more likely that rhamnosylation of the WTA is directly regulated in response to temperature. Although outside of the scope of this study, further research will be needed to elucidate the mechanisms putatively regulating the glycosyl decorations of *Listeria’s* WTA.

### *L. monocytogenes* Can Gain Physiological Refuge from Phage Infection

Physiological refuge can be defined as the ability of a phage-susceptible bacterium to gain transient resistance to phage-infection. The concept was postulated by Lenski (1988) to account for the stability of virulent phage and host populations. Previous studies have provided evidence that bacterial cells can take physiological refuge from phage-infection. For example, a number of studies have shown that adsorption rates of *E. coli* phages are affected by the physiological state of the host (Delbrück, 1940; Hadas et al., 1997; Chapman-McQuiston and Wu, 2008; Høyland-Kroghsbo et al., 2013). The susceptibility of the Firmicutes *Lactococcus* (Mullermerbach et al., 2007) and *Lactobacillus* (Marcó et al., 2015) to phage infection has also been shown to be affected by the physiological state of the host. A specific temperature dependent restriction–modification system has been shown to be present in a clonal group of *L. monocytogenes* strains, which demonstrated a specific case of *L. monocytogenes* taking physiological refuge (Kim et al., 2012). However, we have now shown that temperature generally affects *L. monocytogenes’* susceptibility to phage infection through both mechanisms of adsorption inhibition as well as unidentified internal (i.e., post-adsorption) mechanisms of phage resistance. The data presented here clearly shows that *L. monocytogenes* can gain physiological refuge from phage infection, which should be carefully considered for both the design and implementation of phage-based control and detection applications. These data also offer insight on the evolutionary dynamics of *L. monocytogenes* and its phages, which can help in our understanding of the diversity and transmission of this important foodborne pathogen.

## Author Contributions

TD and JT designed the experiments. MW provided reagents. JT performed the experiments. DK and TD performed the data analyses. TD prepared the figures. TD, JT, DK, and MW interpreted the data. TD and JT wrote the manuscript with valuable feedback from MW and DK.

## Conflict of Interest Statement

MW serves as a scientific advisor for and has a financial interest in Sample6, a company that is producing phage-based diagnostics for foodborne pathogens.
